# Prevalence of acute neurological complications and pathological neuroimaging findings in critically ill COVID-19 patients with and without VV-ECMO treatment

**DOI:** 10.1038/s41598-022-21475-y

**Published:** 2022-10-19

**Authors:** Angelo Ippolito, Hans Urban, Kimia Ghoroghi, Nicolas Rosbach, Neelam Lingwal, Elisabeth H. Adam, Benjamin Friedrichson, Andrea U. Steinbicker, Elke Hattingen, Katharina J. Wenger

**Affiliations:** 1 Institute of Anesthesiology, Intensive Care Medicine and Pain Therapy, University Hospital Frankfurt, Frankfurt, Germany; 2 Institute of Neurology, University Hospital Frankfurt, Frankfurt, Germany; 3 Institute of Neuroradiology, University Hospital Frankfurt, Goethe University, Schleusenweg 2-16, 60528 Frankfurt, Germany; 4 Institute of Diagnostic and Interventional Radiology, University Hospital Frankfurt, Frankfurt, Germany; 5 Institute of Biostatistics and Mathematical Modeling, Goethe University, Frankfurt, Germany

**Keywords:** Medical research, Risk factors

## Abstract

Acute brain injuries such as intracerebral hemorrhage (ICH) and ischemic stroke have been reported in critically ill COVID-19 patients as well as in patients treated with veno-venous (VV)-ECMO independently of their COVID-19 status. The purpose of this study was to compare critically ill COVID-19 patients with and without VV-ECMO treatment with regard to acute neurological symptoms, pathological neuroimaging findings (PNIF) and long-term deficits. The single center study was conducted in critically ill COVID-19 patients between February 1, 2020 and June 30, 2021. Demographic, clinical and laboratory parameters were extracted from the hospital’s databases. Retrospective imaging modalities included head computed tomography (CT) and magnetic resonance imaging (MRI). Follow-up MRI and neurological examinations were performed on survivors > 6 months after the primary occurrence. Of the 440 patients, 67 patients received VV-ECMO treatment (15%). Sixty-four patients (24 with VV-ECMO) developed acute neurological symptoms (pathological levels of arousal/brain stem function/motor responses) during their ICU stay and underwent neuroimaging with brain CT as the primary modality. Critically ill COVID-19 patients who received VV-ECMO treatment had a significantly lower survival during their hospital stay compared to those without (p < 0.001). Among patients treated with VV-ECMO, 10% showed acute PNIF in one of the imaging modalities during their ICU stay (vs. 4% of patients in the overall COVID-19 ICU cohort). Furthermore, 9% showed primary or secondary ICH of any severity (vs. 3% overall), 6% exhibited severe ICH (vs. 1% overall) and 1.5% were found to have non-hemorrhagic cerebral infarctions (vs. < 1% overall). There was a weak, positive correlation between patients treated with VV-ECMO and the development of acute neurological symptoms. However, the association between the VV-ECMO treatment and acute PNIF was negligible. Two survivors (one with VV-ECMO-treatment/one without) showed innumerable microhemorrhages, predominantly involving the juxtacortical white matter. None of the survivors exhibited diffuse leukoencephalopathy. Every seventh COVID-19 patient developed acute neurological symptoms during their ICU stay, but only every twenty-fifth patient had PNIF which were mostly ICH. VV-ECMO was found to be a weak risk factor for neurological complications (resulting in a higher imaging rate), but not for PNIF. Although logistically complex, repeated neuroimaging should, thus, be considered in all critically ill COVID-19 patients since ICH may have an impact on the treatment decisions and outcomes.

## Introduction

The outbreak of Coronavirus disease in 2019 (COVID-19), associated with the severe acute respiratory coronavirus 2 (SARS-CoV-2), quickly became a worldwide public health crisis^1^. While the disease is easily contracted, only a minority of patients with particular comorbidities suffer from severe courses. Typical features of these severe courses are hyper-inflammation combined with acute respiratory distress syndrome (ARDS) and a variety of other complications including neurological manifestations, acute renal injury and bleeding^2,3^. As a consequence, multiple-organ failure (MOF) and death may occur^4–6^. Brain imaging in patients with neurological complications reveals signs of acute ischemic stroke, cerebral venous thrombosis, intracranial hemorrhage (ICH), inflammatory central nervous system (CNS) syndromes (including encephalitis and acute disseminated encephalomyelitis), and posterior reversible encephalopathy syndrome, as well as diffuse leukoencephalopathy with microhemorrhages^7–13^. These brain injuries can be provoked by numerous causes ranging from COVID-19-associated coagulopathy (CAC)^14^, disseminated intravascular coagulation related to sepsis, therapeutic dose anticoagulation^15^, immunologic parainfectious processes^16^, hypoxia and, possibly, also by small-vessel vasculitis^10^. In addition, radiologists have reported emerging evidence of microstructural brain damage from severe courses of COVID-19, even in patients without major stroke or hemorrhagic events^8,10,17–20^. Long-term observations (of 6 months or longer) of survivors who had experienced acute neurological symptoms during their ICU stay, to the best of our knowledge, have not been reported.

As it happens, many of the aforementioned brain injuries are not only associated with COVID-19, but also with veno-venous extracorporeal membrane oxygenation (VV-ECMO) treatment that requires anticoagulation therapy, single cases of heparin overdose, circuit-associated defibrination and thrombocytopenia, disseminated intravascular coagulation, and acquired von Willebrand syndrome^21–24^.

In this article, we report data from an ECMO referral center. Case fatality rate in our critically ill COVID-19 patients treated with VV-ECMO therapy was high with over 60%^25^ compared to less than 40% without the therapy. As reported by others^24^, we clinically noted that acute neurological symptoms in COVID-19 ARDS patients with VV-ECMO were more common than in non-COVID VV-ECMO-treated ARDS cohorts. On the other hand, a similar rate of ICH in patients with ARDS due to COVID-19 compared to other causes of ARDS has been described independently of the VV-ECMO treatment^26^. The purpose of this study was to compare critically ill COVID-19 patients with and without VV-ECMO treatment with regard to acute neurological symptoms and pathological neuroimaging findings (PNIF). We expected that critically ill COVID-19 patients treated with VV-ECMO had an increased risk for developing acute neurological complications and corresponding acute PNIF compared to critically ill COVID-19 patients without an indication for VV-ECMO-treatment. In addition, we invited survivors for a follow-up examination to explore the long-term neurological and MRI findings.

## Material and methods

This single center study was conducted in consecutive, critically ill COVID-19 patients treated at a 1500-bed University Hospital (ECMO referral center) between February 1, 2020 and June 30, 2021. It was a combined analysis of retrospective occurrences of acute neurological symptoms and acute PNIF during the patients’ ICU stay and a prospective analysis of the long-term neurological and imaging findings in survivors who had experienced acute neurological symptoms during their ICU stay.

### Patient cohort

Critically ill patients with confirmed diagnoses of COVID-19 by means of the reverse-transcriptase polymerase chain reaction assay of a nasopharyngeal, throat or lower respiratory tract swab specimen were included. Positive results for COVID-19 were diagnosed by cycle threshold (CT) values below 30. The indications for brain imaging were new-onset neurological changes (e.g., in the level of arousal, brainstem function, motor responses) during the patients’ ICU stays. Neurological changes were evaluated by a neurologist (specialist consultation). The indication and modality of neuroimaging during the patients` ICU stays were the treating physician’s choice and, therefore, independent of this study. Patients with a different primary diagnosis that led to the ICU admission (e.g., ruptured cerebral aneurysm or history of trauma), and COVID-19 as a secondary diagnosis, were excluded. For prospective analysis, survivors who experienced acute neurological symptoms during their ICU stay were invited for an outpatient MRI follow-up examination more than 6 months after the primary occurrence.

### Neuroimaging

Retrospective imaging modalities included brain computed tomography (CT) and magnetic resonance imaging (MRI). COVID-19 ICU patients were examined by brain CT within 24 h if they developed acute neurological symptoms. In cases where neurological symptoms persisted, follow-up imaging was performed (CT and/or MRI). Patients with a CT indication were examined using the spiral CT scanning technique. Routine MRI protocol details are listed in Supplementary Table 1. CT and MRI scans were initially analyzed by radiologists at the institution. The late follow-up MRI protocol used for the prospective analysis is listed in Table 1. Two neuroradiologists (K.W. and E.H. with > 7 and > 25 years of experience, respectively), who were blinded to the clinical features of the patients, reviewed all the examinations (baseline and follow-up) independently; their findings were found to be in consensus. Acute PNIF could be any pathological neuroimaging finding suspected to have developed within hours to days, e.g., acute cerebral infarction and hemorrhage.

**Table 1:** MRI sequences for the late follow-up imaging.

Sequence	Plane	Slice thickness (mm)	Pixel spacing	TR; TE; Number of Averages	Comment
T2W TSE	Axial	5	0.53	4980; 92; 3	
FLAIR	Axial	4	0.69	8500; 81; 1	
SWI	Axial	12	0.86	27; 20; 1	
T2*W	Axial	5	0.43	631; 19.9; 1	
DWI/ADC	Axial	5	0.57	3800; 95; 3	b-values 0, 500, 1000
DWI/ADC	Coronal	5	0.57	3900; 112; 2	b-values 0, 500, 1000
Arterial TOF-MRA	Axial	0.5	0.26	21; 3,42; 1	
3D FLAIR	Sagittal	0.9	0.45	5000; 387; 1	Optional

*T2W* T2-weighted, *FSE* fast spin echo, *TSE* turbo spin echo, *FLAIR* fluid-attenuated inversion recovery, *SWI* susceptibility weighted imaging, *DWI* diffusion-weighted imaging, *ADC* apparent diffusion coefficient, *TOF-MRA* time-of-flight MR-angiography.

### Clinical and laboratory data

Structured data (laboratory findings) were extracted from fixed-mode databases with the help of the Department of Information and Communications Technology of the University Hospital, Frankfurt (DICT). Unstructured data were extracted manually from the hospital information system (HIS). The data protection concept for the extracted data was approved by the local data protection officer and in compliance with the General Data Protection Regulation (GDRP). Clinical and laboratory data were reviewed by an anesthesiologist (A.I. with > 8 years of experience). We recorded the age and sex of the patient, their past medical history, days on invasive mechanical ventilation (IVM) and VV-ECMO at the time point of neuroimaging and during the hospital stay, therapeutic dose anticoagulation (unfractionated heparin (UFH) or low-molecular-weight heparin (LMWH)) or overt disseminated intravascular coagulation at the time point of neuroimaging, laboratory findings at the time point of neuroimaging, and death of the patient during their in-hospital stay. Anticoagulation strategies were targeted at the following laboratory parameter ranges: activated partial thromboplastin time (aPTT) range 40–60 s for treatment with UFH and anti-factor Xa activity (aXa) 0.4–1.0 U/mL for treatment with LMWH (enoxaparin). Next to absolute values, both parameters were reported as being in, above or below the target range (aXa rounded to one decimal).

### Neurological examination

Survivors who experienced acute neurological symptoms during their ICU stay were subjected to a neurological examination. This was reported in a structured approach and included the following major areas: cranial nerves including the visual system, muscle strength, tone and bulk, reflexes, coordination and sensory function. The rehabilitation and functional potential of former critically ill patients was assessed using the Barthel Index of Activities of Daily Living. The Barthel-Scale was developed to assess the rehabilitation and functional potential of patients with neurovascular events. Several studies have proven its efficacy, validity and reliability in this setting, especially in young patients (< 65 years) with no prior cognitive impairment^27–29^. The degree of disability or dependence in daily activities was recorded using the Modified Rankin Scale (mRS) which was developed for stroke patients^30,31^. General cognitive functioning was measured using the Mini-Mental State Examination (MMSE) test^32,33^. The neurological baseline status prior to the patient’s ICU stay could be determined by the patient’s self-report or the report from a separate party who was familiar with the patient’s abilities (for example, a relative).

### Statistical analysis

Data were described using means ± standard deviations (rounded to integers if not otherwise specified). Categorical variables were compared using the Pearson χ^2^ test or Fisher exact test, as appropriate. The Phi coefficient was used to understand the strength of the relationship between two variables. For ordinal data, the Wilcoxon-Mann–Whitney U-Test was employed. Continuous data was log-transformed and tested for normal distribution. Since the data were not normally distributed, the Wilcoxon-Mann–Whitney U-test was performed. The Kaplan–Meier estimator was used to estimate the survival function. The starting-point for statistical analysis was ICU admission and the endpoint was ICU discharge. P-values < 0.05 were considered statistically significant. Analysis was performed using JASP (Version 0.16) and BiAS (Version 11.12).

### Ethics approval

This study was approved by the ethical committee of the University Hospital, Frankfurt (Nr. 20-643) and was in accordance with the 1964 Helsinki Declaration and its later amendments.

### Consent to participate

The requirement for written informed consent was waived by the ethical committee of the University Hospital, Frankfurt (Nr. 20-643) for the observational part of the study in these critically ill patients. Written informed consent for follow-up examinations was obtained from survivors.

## Results

### Whole population (ICU cohort)

**Figure 1:**
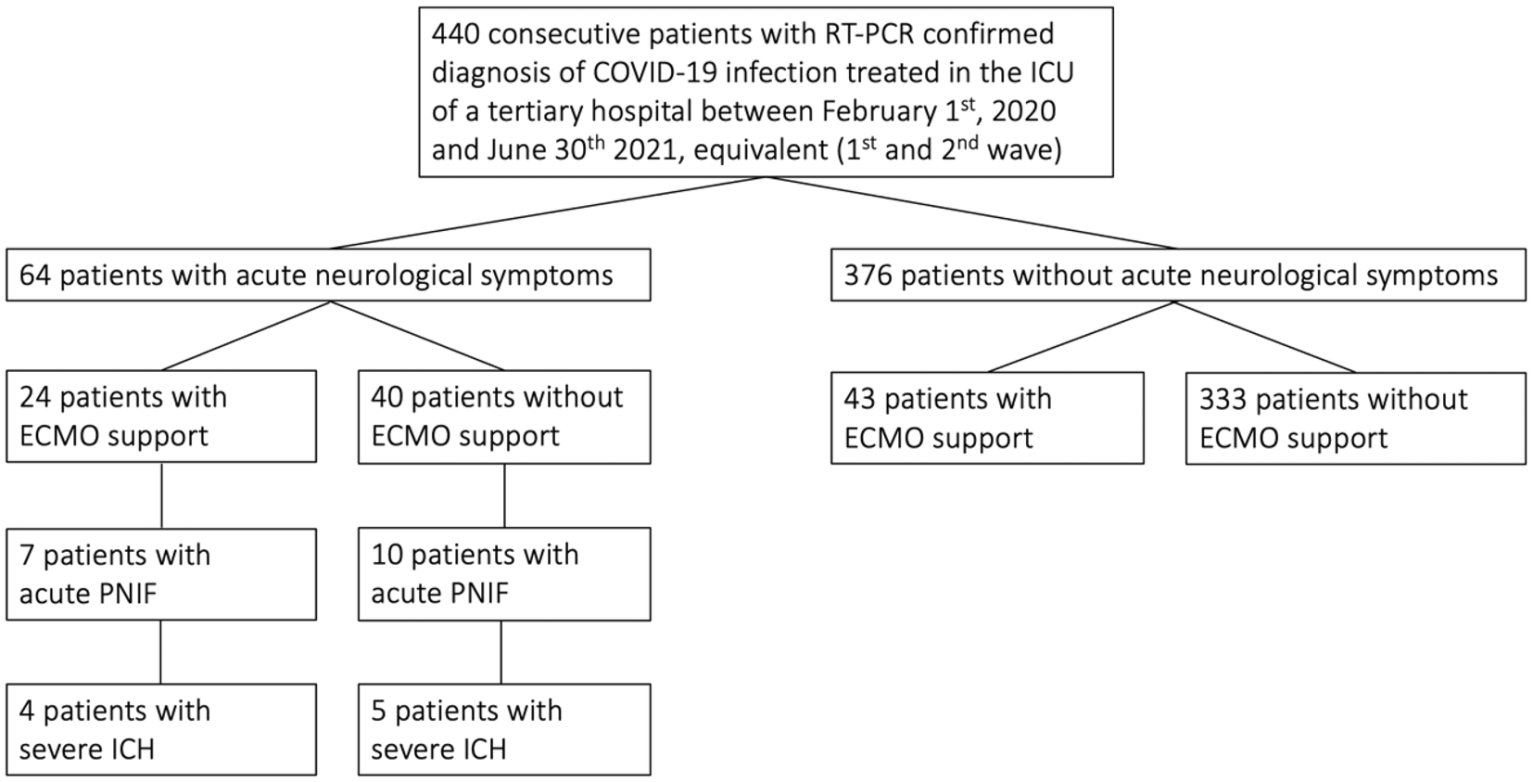
Flow chart of the patients included in the study.

A total of 440 patients with COVID-19 as their primary diagnosis were hospitalized in the ICU between February 1, 2020 and June 30, 2021, equivalent to the so-called 1st and 2nd waves. The study site is an ECMO referral center. The patient flow chart is shown in Fig. 1. Patient characteristics are summarized in Table 2.

**Table 2:** Patient characteristics including subpopulations.

	Whole population (n = 440)	Subpopulation with VV-ECMO treatment (n = 67)	Subpopulation without VV-ECMO-treatment (n = 373)	Subpopulation with acute neurological symptoms leading to neuroimaging (n = 64)	Subpopulation with acute PNIF (n = 17)
Age (years mean, SD)	62 ± 15	54 ± 12	64 ± 15	61 ± 14	61 ± 15
% female	23	10	26	14	18
Prior conditions (mean, range)	2 (0–8)	1.5 (0–6)	2 (0–8)	2 (0–8)	2 (0–5)
Prior conditions specified	HTN (46%), CKD (17%), CLD (4%), CHD (9%), CPD (20%), CCD (9%), DM (32%), MAL (8%), OB (26%), DEM (3%), NIC (7%)	HTN (33%), CKD (9%), CLD (5%), CHD (9%), CPD (18%), CCD (2%), DM (21%), MAL (3%), OB (39%), DEM (0%), NIC (16%)	HTN (48%), CKD (18%), CLD (4%), CHD (32%), CPD (21%), CCD (10%), DM (34%), MAL (8%), OB (24%), DEM (3%), NIC (6%)	HTN (36%), CKD (20%), CLD (8%), CHD (30%), CPD (31%), CCD (3%), DM (28%), MAL (11%), OB (38%), DEM (0%), NIC (8%)	HTN (41%), CKD (18%), CLD (6%), CHD (24%), CPD (18%), CCD (6%), DM (18%), MAL (6%), OB (35%), DEM (0%), NIC (6%)
% ECMO; mean duration days, SD	15; 28 ± 22	100; 28 ± 22	0	At the time point of Neuroimaging 38; 13 ± 14	At the time point of Neuroimaging 50; 5 ± 3
% IVM; mean duration days, SD	59; 21 ± 19	100; 36 ± 24	42; 15 ± 12	At the time point of Neuroimaging 88; 16 ± 14	At the time point of Neuroimaging 82; 13 ± 11
%RRT	12	19	10	48	65
Mean length of stay on ICU, SD	15 ± 17	15 ± 17	11 ± 10	Up to the time point of Neuroimaging 13 ± 13	Up to the time point of Neuroimaging 13 ± 11
% Death during hospital stay	38	64	34	53	65

*HTN* hypertension, *CKD* chronic kidney disease, *CLD* chronic liver disease, *CHD* chronic heart disease, *CPD* chronic pulmonary disease, *CCD* chronic cerebro-vascular disease, *DM* diabetes, *MAL* malignancy, *OB* obesity defined by body mass index (BMI; kg/m^2^) ≥ 30, *DEM* clinically proven dementia, *NIC* smoking. Smoking is likely to be underreported.

**Figure 2:**
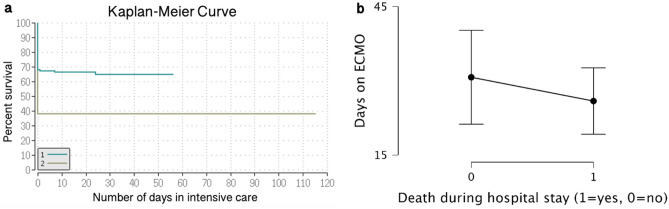
Covid-19 patient survival with and without VV-ECMO intervention during their hospital stay. (**a**) The Kaplan–Meier estimator was used to estimate survival function. The starting-point was ICU admission and the endpoint was ICU discharge. Group 1 = critically ill COVID-19 patients treated without VV-ECMO; Group 2 = critically ill COVID-19 patients with VV-ECMO treatment. Critically ill COVID-19 patients with VV-ECMO treatment had a significantly lower survival during the hospital stay compared to those without the treatment (p < 0.001). (**b**) Among the VV-ECMO patients there was no significant difference with regard to the number of days on VV-ECMO between the patients who died during their hospital stay and survivors (p = 0.35). The descriptive plot of means and error bars indicate the traditional 95% confidence intervals.

The case fatality rate (CFR) during hospital stays was 38% (168 patients) for the entire COVID-19 cohort. Fifty-nine percent of the invasively mechanically ventilated patients and 64% of patients treated with VV-ECMO (43 and 67 patients, respectively) died during their hospital stay. Critically ill COVID-19 patients who were treated with VV-ECMO had a significantly lower survival during their hospital stay compared to those without the treatment (p < 0.001); the corresponding Kaplan–Meier Curve is shown in Fig. 2. Among the VV-ECMO patients, there was no significant difference with regard to the number of days on VV-ECMO between the patients who died during their hospital stay and survivors (p = 0.35; Fig. 2).

### Subpopulation with VV-ECMO treatment

67 patients (15%) received VV-ECMO treatment at our ECMO referral center for a mean duration of 28 ± 22 days. 72% of patients treated with VV-ECMO were referred in a critical condition from another hospital to our University Hospital. After initial recovery, VV-ECMO had to be reimplanted in one patient. Two patients were transferred with VV-ECMO to a tertiary care center closer to their hometown. VV-ECMO patients were significantly younger than those who did not receive this therapy (p < 0.001; mean with VV-ECMO 54 ± 12 years; mean without VV-ECMO 64 ± 15 years) and 90% were male. There were significantly more obese patients in the subpopulation with VV-ECMO treatment (p < 0.01; 39% vs. 24%). Patient characteristics are summarized in Table 2.

Twenty-four of 67 patients treated with VV-ECMO developed acute neurological symptoms. Hypercapnic acidosis (pH < 7.25 with PaCO2 > 60) was present in 37,5% of cases before ECMO initiation. In the first 24 h after ECMO initiation, patients showed a median relative change in PaCO2 of − 28.8% (the largest relative decrease in PaCO2 in the first 24 h after ECMO initiation was − 44%).

### Subpopulation with acute neurological symptoms

Sixty-four patients (15%) developed acute neurological symptoms during their ICU stay and underwent neuroimaging. The symptoms were documented as pathological levels of arousal (41 patients, 64%), pathological brain stem function (21 patients, 33%) and pathological motor responses (2 patients, 3%). Of the patients with newly developed pathological motor responses, one patient presented with a unilateral neglect and hemiparesis and one with sensorimotor deficits of both legs (CT ordered to rule out an intracranial abnormality with elevated intracranial pressure prior to lumbar puncture). Patient characteristics are summarized in Table 2.

**Figure 3:**
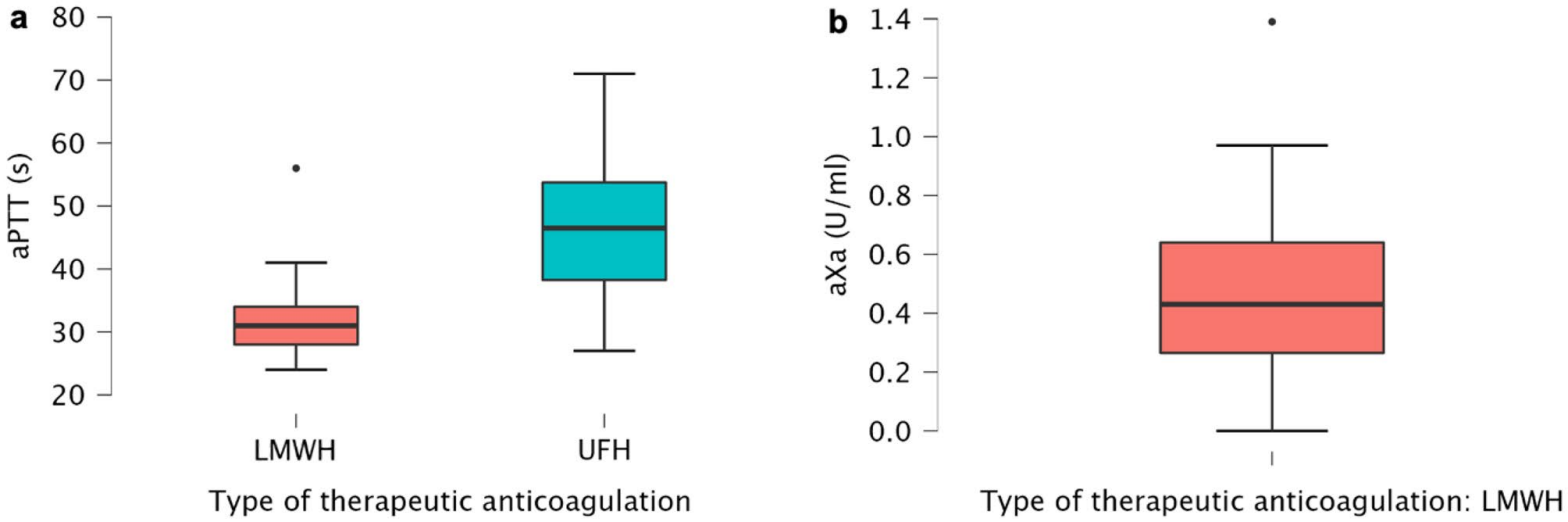
Box and whisker plots with min., max., 25th, 50th (median) and 75th percentiles and outliers. (**a**) aPTT (s) and (**b**) aXa (U/ml) levels of patients treated with therapeutic dose anticoagulation (UFH, LMWH = enoxaparin) at the time point of neuroimaging.

None of the patients suffered from overt disseminated intravascular coagulation based on the International Society on Thrombosis and Hemostasis diagnostic scoring system^34^. Sixty-one patients (95%) received therapeutic dose anticoagulation. The aPTT mean was 46 ± 12 s for treatment with UFH, while the aXa mean was 0.5 ± 0.3 U/mL for treatment with NMH. A box and whisker plot is shown in Fig. 3. While there was no significant relationship between the ICH detected on the brain CT (or if the symptoms persisted and were not explained by the further brain CT and/or brain MRI scans) and the patients with therapeutic dose anticoagulation above the target range (p = 0.11 for ICH of any severity and p = 0.42 for severe ICH), we did find a weak, positive correlation between cerebral ischemia and the therapeutic dose anticoagulation below the target range (p < 0.01; Phi coefficient 0.35). There was no significant difference between the patients with and without ischemia or ICH (of any severity) with regard to the following laboratory parameters: Platelet count (/nl), C-reactive protein level (mg/dl) and hemoglobin level (g/dl). Respective box whisker plots are shown in Supplementary Fig. 1. D-dimer and fibrinogen levels were not available in all cases at the time point of neuroimaging.

### Subpopulation with acute pathological neuroimaging findings (PNIF)

In cases where the neurological symptoms persisted and were not explained by the initial CT, follow-up imaging was performed (CT or MRI). Thirteen patients (20%) had follow-up CTs and 5 patients had follow-up MRIs (8%). Seventeen patients (27%) with acute neurological symptoms showed acute PNIF in one of the imaging modalities during their ICU stay. The subpopulation’s CFR during hospital stay was 65% (11 patients). All of these died from their neurological complications after a mean of 5 ± 8 days. Patient characteristics are summarized in Table 2. Acute neurological symptoms that led to neuroimaging and neuroimaging findings are summarized in Supplementary Table 2.

**Figure 4:**
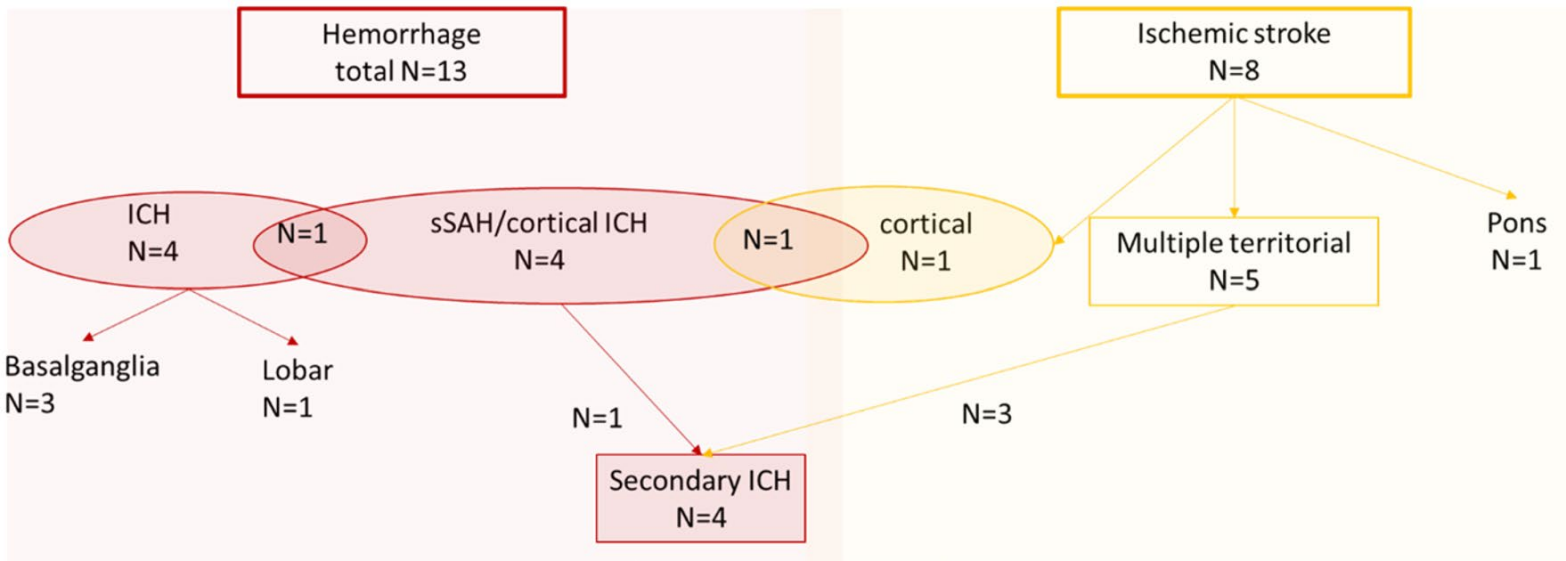
Flow chart of the neuroimaging findings. Sixty-four patients (15%) developed acute neurological symptoms during their ICU stay and underwent neuroimaging. Among those patients, 17 (27%) showed acute PNIF in one of the imaging modalities during their ICU stay.

**Figure 5:**
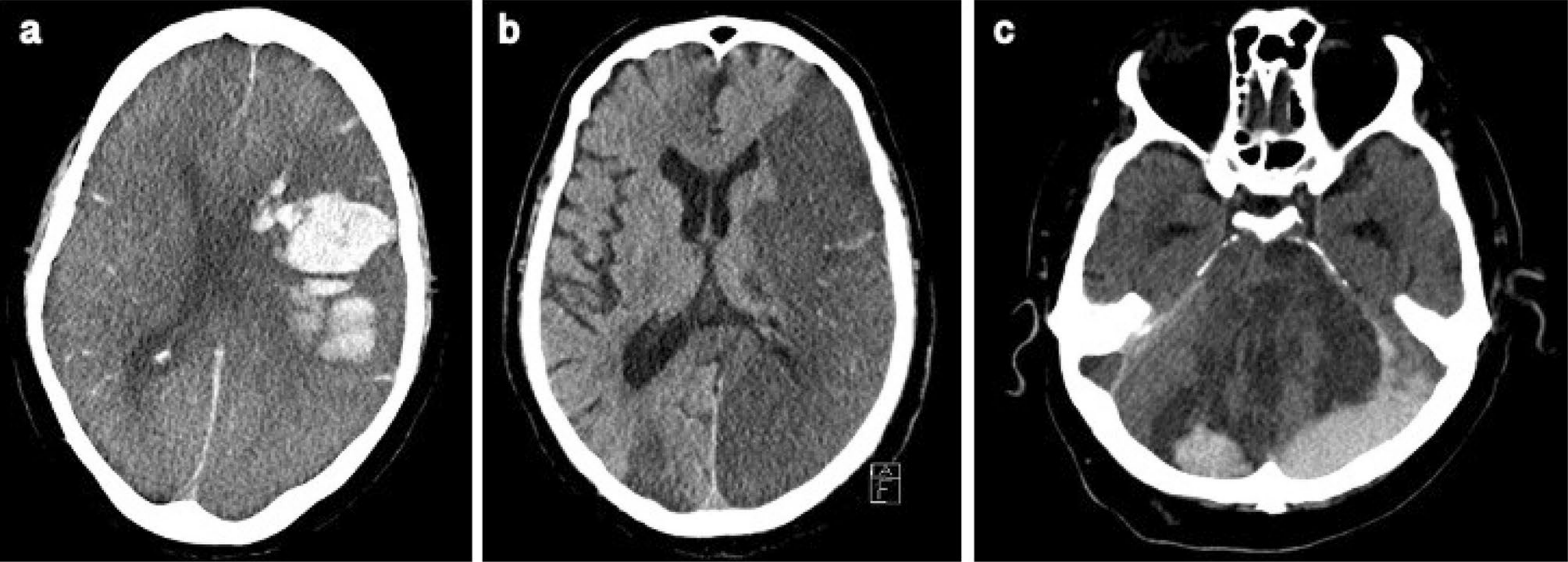
Acute PNIF on Brain CT in three exemplary cases. (**a**) Case 1. Lobar hemorrhage in the left hemisphere and global subarachnoid hemorrhage with secondary intraventricular hemorrhage. (**b**) Case 8. Multiple, presumably embolic, infarcts in multiple cerebral circulations (left MCA, right ACA, PCA bilateral, cerebellum). (**c**) Case 12. Intracranial hemorrhage after multiple infarcts (PCA bilateral, brainstem and cerebellum). CT angiography was not performed in this fatal case but the underlying cause was assumed to be basilar artery occlusion.

In 6/17 (35%) patients the initial CT scan was negative, however, the follow-up imaging did reveal pathologies; five of these cases were detected by follow-up CT while one was detected by MRI (small cortical infarct, Case 14). Thirteen patients suffered from ICH (9 severe, 4 mild) and 8 patients suffered from acute cerebral infarction; among those patients, 4 suffered from secondary ICH. These findings are summarized in Fig. 4. Exemplary imaging findings are shown in Fig. 5. In our entire ICU cohort, we found a weak, positive correlation between patients treated with VV-ECMO and the development of acute neurological symptoms (p < 0.001; Phi coefficient 0.26). However, there was no substantively significant association between the VV-ECMO treatment and acute PNIF on the brain CT or, if the symptoms persisted and were not explained by the initial CT, further brain CT and/or brain MRI scans (p < 0.01; Phi coefficient 0.13).

### Follow-up examination of survivors who experienced acute neurological symptoms during their ICU stay

Of the 27 survivors, 10 patients (37%) agreed to participate in an outpatient follow-up examination. One patient was excluded due to contraindications for MRI examination (pacemaker). Seventeen patients either had non-traceable changes of contact information or were too impaired to participate (i.e., living in a nursing home). The mean interval between the acute neurological findings and the late follow-up examination was 303 ± 117 days. The demographic and clinical parameters as well as the performance scores and late follow-up neuroimaging findings are summarized in Supplementary Table 3. The mean loss of function from the baseline to the long-term follow-up was 5 ± 10 points on the Barthel scale (rounded to 5-point increments) and 1 ± 1 point on the modified Rankin Scale (mRS). The mean MMSE score at follow-up was 26 ± 3. Two patients (one with VV-ECMO treatment, one without) showed innumerable, punctuate microhemorrhages that predominantly involved the juxtacortical white matter (cases presented in the “Supplement”). All other patients showed few or no microhemorrhages. However, those patients having only a few microhemorrhages were elderly, thus, our findings could not be distinct from age-related microbleeds. None of the patients showed diffuse leukoencephalopathy, described in literature as being symmetric and confluent with mild restricted diffusion involving bilateral deep and subcortical white matter^10,35,36^.

## Discussion

In this study we describe a single center ICU cohort of 440 patients with COVID-19 as their primary diagnosis. Of the 440 patients, 67 patients received VV-ECMO treatment (15%). Overall, 64 patients developed acute neurological symptoms (documented as a pathological level of arousal, pathological brain stem function and pathological motor responses) during their ICU stay, serving as a trigger for neuroimaging with brain CT as the primary modality. Of those 64 patients, 24 patients (38%) required VV-ECMO treatment at the time point of neuroimaging.

In our entire ICU cohort, there was a weak, positive correlation between patients treated with VV-ECMO and the development of acute neurological symptoms. However, the association between acute PNIF on the brain CT (or if the symptoms persisted and were not explained by the further brain CT and/or brain MRI scans) and the VV-ECMO treatment was negligible. We offer two plausible explanations for this. First, the interpretation of neurological signs in critically ill comatose patients is often confounded by sedation, neuromuscular blockade, pain, delirium, anxiety, metabolic and physiological disturbances, and the physical limitations caused by intubation and ECMO treatment^37^. Second, COVID-19-associated diffuse leukoencephalopathy, ECMO or COVID-19-associated microhemorrhages or small embolic/brain stem infarcts that influence a patient’s neurological status might not be visible on CT imaging, especially in the early stages^10^. While intracranial (macro)hemorrhage is easily diagnosed by brain CT^38,39^, the millimeter-sized paramagnetic blood products of microhemorrhages are best visualized with gradient-echo MRI^40^. However, the European Society of Intensive Care Medicine (ESICM) expert panel considers CT a reasonable initial imaging modality for the evaluation of patients with focal neurological deficits or unexplained depression of consciousness, when the need for continuing organ support makes MRI logistically difficult^37^. Ultimately, neurological injuries in adults treated with VV-ECMO and those related to COVID-19 cannot be readily distinguished. Mateen et al. examined neurological injuries in adults treated with ECMO due to various precipitating factors and found that neurological events affect at least half of those patients and may be predicted by higher age and lower nadir arterial oxygen pressure. Diagnoses included subarachnoid hemorrhage, ischemic watershed infarctions, hypoxic-ischemic encephalopathy, unexplained coma and brain death^41^. At the same time, acute ischemic stroke, cerebral venous thrombosis, cerebral hemorrhage, as well as diffuse leukoencephalopathy with microhemorrhages, have been reported with COVID-19-related causes^7–11,13^.

Another important aspect that should be considered as a possible cause of an increased incidence of neurological complications in patients receiving VV-ECMO for respiratory failure is the magnitude of Pa_CO2_ correction upon ECMO initiation. Previous studies described the rapid pCO2 decrease at ECMO initiation (> 50%) as an important aspect that may lead to increased incidence of neurological complications^21,42^. However, at referral sites, physicians are trained to avoid this effect by limiting ECMO flow to adjust for extreme pCO2 changes. In our study, patients with VV-ECMO and development of acute neurological symptoms (24 patients) showed a median relative change in PaCO2 of − 28.8% (the largest relative decrease in PaCO2 in the first 24 h after ECMO initiation was − 44%).

Among those patients treated with VV-ECMO, 10% showed acute PNIF in one of the imaging modalities during their ICU stay (vs. 4% of patients in the overall COVID-19 ICU cohort). Furthermore, 9% showed primary or secondary ICH of any severity (vs. 3% overall), 6% severe ICH (vs. 1% overall), 6% cerebral infarctions with or without secondary ICH (vs. 1.8% overall) and 1.5% non-hemorrhagic cerebral infarctions (vs. < 1% overall). Our rate of cerebral infarctions with or without secondary ICH is slightly higher than that recently reported by Qureshi et al. in a multicenter cohort of 27,676 patients (1.8 vs. 1.3%), although they reported on all in-hospital events, not just the ICU cohort^43^. A review article by Wang et al. describes reported rates ranging from 2.5 to 5%, again for all hospitalized patients^44^. Leasure et al. describe a prevalence of spontaneous intracerebral hemorrhage of 0.2% in a registry of 21,483 hospitalized patients (approximately 30% of these were in intensive care)^45^. Again, our rate in critically ill patients is higher, but is not nearly as high as that reported by Lang et al., where 19% of COVID-19 ICU patients had moderate or severe ARDS (with or without VV-ECMO)^26^. One explanation for this discrepancy could be our lower imaging rate (15% overall; 36% for VV-ECMO patients vs. 55% with or without VV-ECMO). With regard to the ICH of any severity in COVID-19 patients with VV-ECMO treatment, our rate is considerably lower than that previously reported by Seeliger et al. (18), even if we account for the one patient with microbleeds detected only on the late follow-up MRI (10% vs. 20%). Furthermore, in this case, our imaging rate was lower (36 vs. 55% for patients treated with VV-ECMO) than Seeliger et al.’s. Currently, our institution does not perform routine screening Brain CT scans in patients without neurological symptoms due to the exposure to radiation and potential risks associated with the transportation of critically ill patients. However, sedatives and muscle relaxants used in critically ill patients can mask symptoms of brain injury, especially in minor events such as small cortical or sulcal hemorrhages. Several studies have reported ECMO-associated ICH diagnoses that were made in the absence of neurological symptoms^46–49^. In addition, initial scans were negative in 35% of our patients although later follow-up imaging (mostly CT) did reveal pathologies.

While there was no significant relationship between the ICH detected on neuroimaging during the ICU stay and therapeutic dose anticoagulation above or below the target range, we did find a weak, positive correlation between cerebral ischemia and therapeutic dose anticoagulation below the target range. Although the pathogenesis of hypercoagulability in COVID-19 is incompletely understood, arterial thrombotic events occur and anticoagulation might be effective in preventing these events. To address the potential of anticoagulation in the prevention of such thrombotic events, large-scale, prospective, multicenter, randomized clinical trials are presently ongoing^50^.

One finding that stands out with regard to our follow-up examination cohort is that none of the 9 formerly critically ill COVID-19 patients with acute neurological symptoms during their ICU stay showed abnormal white matter T2w-hyperintensities (indicative of diffuse leukoencephalopathy) on their long-term follow-up MRI scans. Two of these patients had already undergone an MRI scan during their ICU stay showing no restricted diffusion or T2w-hyperintensities. Seven of them had undergone Brain CT scans only where subtle changes of white matter might have been obscured. Two of the 9 patients showed numerous punctate microhemorrhagic foci on follow-up MRI which, in the few case series reported in literature, often presented combined with leukoencephalopathy^10,51,52^. This raises, at least, the possibility that the cytotoxic edema described in critically ill COVID-19 patients in the literature could be reversible.

Limitations of the current report include the small sample size of ECMO patients and the partially retrospective nature without neuroimaging prior to the ICU stay. Another limitation is the patient’s self-report or report from a separate party who were familiar with the patient’s abilities (such as a relative) with regard to their neurological baseline status.

## Conclusion

To conclude, our study indicates that about 15% of critically ill COVID-19 patients may develop acute neurological symptoms during their ICU stay. These neurological symptoms serve as a trigger for neuroimaging at our institution. Currently, we do not perform routine screening using brain CT scans in non-symptomatic ICU patients due to the exposure to radiation and potential risks associated with the transportation of critically ill patients. VV-ECMO was found to be a weak risk factor for neurological symptoms, however, the association of VV ECMO with actual PNIF was statistically negligible. In our cohort, about 75% of COVID-19 patients with acute neurological symptoms had normal brain CT findings. However, comparing our rate of primary or secondary ICH of any severity (3% overall, 9% of patients treated with VV-ECMO) to similar collectives with higher imaging rates, these complications are likely to be underreported. In addition, initial scans were negative in 35% of patients but follow-up imaging (mostly CT) revealed pathologies. Independent of the VV-ECMO treatment, a small group of patients presented with innumerate microbleeds on long-term follow-up MRI with corresponding neurological deficits. Our study included only a small number of patients treated with VV-ECMO and is, therefore, only suitable for the generation of hypotheses. The data suggest that repeated neuroimaging might be considered in all COVID-19 patients during their ICU stay since intracranial hemorrhages may have an impact on further treatment decisions and the patients’ neurological outcomes.

## Supplementary Information

Supplementary Information.

## Data Availability

The data that support the findings of this study are available from the corresponding author, KJW, upon reasonable request.

## Abbreviations

ADC: Apparent diffusion coefficient
aPTT: Activated partial thromboplastin time
ARDS: Acute respiratory distress syndrome (ARDS)
aXa: Anti-factor Xa activity
BMI: Body mass index
CAC: COVID-19-associated coagulopathy (CAC)
CFR: Case fatality rate
CNS: Central nervous system (CNS)
COVID-19: Coronavirus disease 2019
CT: Computed tomography and cycle threshold
DICT: Department of Information and Communications Technology of the University Hospital Frankfurt
DWI: Diffusion-weighted imaging
ECMO: Extracorporeal membrane oxygenation
ESICM: European Society of Intensive Care Medicine
FLAIR: Fluid-attenuated inversion recovery
FSE: Fast spin echo
GDRP: General Data Protection Regulation
HIS: Hospital information system
ICH: Intracranial hemorrhage (ICH)
IRB: Institutional review board (IRB)
IVM: Invasive mechanical ventilation
LMWH: Low-molecular-weight heparin
MMSE: Mini-Mental State Examination
MOF: Multi-organ failure (MOF)
MRI: Magnetic resonance imaging
PNIF: Pathological neuroimaging findings
RRT: Renal replacement therapy
SWI: Susceptibility weighted imaging
TOF-MRA: Time-of-flight MR-angiography
TSE: Turbo spin echo
T2W: T2-weighted
UFH: Unfractionated heparin
VV: Veno-venous
