# Supplementary material for: Prevalence of acute neurological complications and pathological neuroimaging findings in critically ill COVID-19 patients with and without VV-ECMO treatment
Source: Sci Rep. 2022 Oct 19;12:17423. doi: 10.1038/s41598-022-21475-y (PMC9579632; doi:10.1038/s41598-022-21475-y)
Supplement: Supplementary file 1 — Supplementary Information. [file 41598_2022_21475_MOESM1_ESM.docx]

Supplement

| **Sequence** | **Plane** | **Slice thickness (mm)** | **Pixel spacing** | **TR; TE; Number of Averages** | **Comment** |
| --- | --- | --- | --- | --- | --- |
| TIRM dark fluid | axial | 5 | 0.75 | 8000; 79; 2 |  |
| T1W SE | axial | 5 | 0.78 | 400; 5; 1 |  |
| T2W TSE | axial | 5 | 0.56 | 4470; 91; 2 |  |
| arterial TOF-MRA | axial | 0.5 | 0.35 | 23; 7; 1 |  |
| RESOLVE (DWI/ADC) | axial | 5 | 1.56 | 4690; 66, 1 | b-values 0, 800 |
| RESOLVE (DWI/ADC) | coronal | 5 | 1.44 | 4690; 66; 1 | b-values 0, 800 |
| T2*W | axial | 5 | 0.49 | 884; 25; 1 |  |
| T1W MPRAGE CE | axial | 0.9 | 0.98 | 1680; 2.61; 1 |  |
| T1W SE CE | axial | 5 | 0.75 | 400; 5; 1 |  |

**Supplementary table 1** MRI sequences for neuroimaging during ICU stay acquired at a 1.5 T clinical scanner. TIRM=turbo inversion recovery magnitude; T1W=T1-weighted; T2W=T2-weighted; SE=spin echo; TSE=turbo spin echo; TOF-MRA=time-of-flight MR-angiography; RESOLVE=readout segmentation of long variable echo trains (approach for obtaining high-resolution DWI images); CE=contrast enhanced (gadovist 0.1 mmol per kilogram body weight)

| **Case** | **Age/Sex** | **Past medical history** | **Acute neurological symptoms leading to neuroimaging** | **Acute PNIF** | **Severity of ICH** | **Days on IMV** | **Days from ECMO cannulation to PNIF** | **Death during hospital stay** | **Therapeutic dose anti-coagulation** | **Anticoagulation in, above, below target range** |
| --- | --- | --- | --- | --- | --- | --- | --- | --- | --- | --- |
| 1 | 63/m |  | Pathological brain stem function | Lobar hemorrhage left hemisphere and global subarachnoid hemorrhage with secondary intraventricular hemorrhage | severe | 17 | N/A | 1 | 1 | in |
| 2 | 62/m | CHD | Pathological brain stem function | Basal ganglia/thalamic hemorrhage left hemisphere with secondary intraventricular hemorrhage | severe | 27 | N/A | 1 | 1 | in |
| 3 | 61/m | HTN | Pathological level of arousal | Basal ganglia/thalamic hemorrhage left hemisphere with secondary intraventricular hemorrhage | severe | 10 | N/A | 1 | 0 |  |
| 4 | 66/m | HTN, OB, NIC | Pathological brain stem function | Atraumatic sulcal subarachnoid hemorrhage right frontal lobe | mild | 16 | 5 | 1 | 1 | above |
| 5 | 57/m | HTN, NIC | Pathological level of arousal | Small cortical hemorrhage | mild | 5 | 5 | 0 | 1 | in |
| 6 | 33/m | OB, CPD, DM | Pathological brain stem function | Paramedian pontine infarction right side | N/A | 0 | 0 | 1 | 1 | in |
| 7 | 70/m | HTN | Pathological brain stem function | Bilateral basal ganglia hemorrhage with secondary intraventricular hemorrhage | severe | 15 | N/A | 1 | 1 | below |
| 8 | 83/m | HTN, CHD, DM | Pathological level of arousal | Multiple infarcts in multiple cerebral circulations (left MCA, right ACA, PCA bilateral, cerebellum) | N/A | 0 | N/A | 1 | 1 | below |
| 9 | 62/m | HTN, CPD | Pathological brain stem function | Intracranial hemorrhage after multiple infarcts (right MCA, PCA) | severe | 2 | 1 | 1 | 1 | below |
| 10 | 70/m | HTN, CHD, CPD, DM, NIC | Pathological level of arousal | Atraumatic sulcal subarachnoid hemorrhage right frontal lobe and both parietal lobes, focal cortical hemorrhage of the right postcentral gyrus | mild | 15 | N/A | 0 | 1 | in |
| 11 | 28/m |  | Pathological brain stem function | Initial atraumatic sulcal subarachnoid hemorrhage both frontal and parietal lobes, two days later right frontal lobar hemorrhage | severe | 16 | 8 | 1 | 1 | in |
| 12 | 51/m |  | Pathological brain stem function | Intracranial hemorrhage after multiple infarcts (PCA bilateral) and cerebellum | severe | 20 | 8 | 1 | 1 | below |
| 13 | 82/m | HTN | Pathological level of arousal | Lobar hemorrhages right frontal and occipital lobes | severe | 38 | N/A | 0 | 1 | in |
| 14 | 63/m | HTN, CHD, OB | Pathological level of arousal | Small cortical infarct (right MCA) | N/A | 7 | N/A | 0 | 1 | below |
| 15 | 77/m | CHD | Pathological level of arousal | Multiple infarcts in multiple cerebral circulations (right MCA, left PCA and cerebellar) | N/A | 0 | N/A | 0 | 1 | below |
| 16 | 67/m |  | Pathological brain stem function | Sulcal subarachnoid hemorrhage right frontal and parietal lobe, small cortical infarct (right MCA) | mild | 24 | N/A | 0 | 1 | above |
| 17 | 48/m | HTN, CLD, OB | Pathological brain stem function | Intracranial hemorrhage after multiple infarcts (ACA, MCA, PCA bilateral) and cerebellum | severe | 4 | 1 | 1 | 1 | below |

**Supplementary table 2** Patients with acute pathological neuroimaging findings (PNIF) during their ICU stay. Binary variables: 0=no, 1=yes; Days on IMV to acute pathological neuroimaging findings. Abbreviations: CHD=chronic heart disease; HTN=hypertension; OB=obesity; NIC=nicotine; DM=diabetes mellitus; CPD=chronic pulmonary disease; CLD=chronic liver disease; ACA=anterior cerebral artery; MCA=middle cerebral artery; PCA=posterior cerebral artery; N/A=not applicable, patients not on VV-ECMO during neuroimaging

| **Case** | **Age/Sex** | **Past medical history** | **Acute neurological symptoms leading to neuroimaging** | **Acute PNIF** | **Overall days on IMV until discharge** | **Overall days on ECMO until discharge** | **Therapeutic dose anti-coagulation** | **Anticoagulation in, above, below target range** | **Microbleeds/siderosis late follow-up MRI severe, non-age-related, non-hypertensive/CAA pattern** | **Loss of score on Barthel scale baseline to long-term follow-up** | **Loss of function on modified Rankin Scale (mRS) baseline to long-term follow-up** |
| --- | --- | --- | --- | --- | --- | --- | --- | --- | --- | --- | --- |
| 1 | 51/m | DM | Pathological level of arousal | - | 32 (VOD) | N/A | 1 | below | 0 | 30 | 1 |
| 2 | 63/m | NIC | Pathological brain stem function | - | 71 (VOD) | 66 | 1 | in | 0 | 20 | 3 |
| 3 | 32/m | DM | Pathological level of arousal | - | 9 | N/A | 1 | in | 0 | 0 | 0 |
| 4 | 71/f | HTN, CHD, OB | Pathological motor responses | - | 0 | N/A | 1 | above | 0 | 5 | 1 |
| 5 | 77/m |  | Pathological brain stem function | - | 25 | N/A | 1 | above | 0 | 0 | 0 |
| 6 | 70/m | HTN, CHD, CPD, DM, NIC | Pathological level of arousal | Atraumatic sulcal subarachnoid hemorrhage right frontal lobe and both parietal lobes, focal cortical hemorrhage of the right postcentral gyrus | 40 (VOD) | N/A | 1 | in | 1 | 0 | 2 |
| 7 | 59/f | CPD | Pathological level of arousal | - | 0 | N/A | 1 | in | 0 | 0 | 0 |
| 8 | 55/m | CDP | Pathological level of arousal | - | 27 | 18 | 1 | below | 0 | 10 | 2 |
| 9 | 40/m |  | Pathological level of arousal | - | 44 (VOD) | 7 | 1 | in | 1 | 0 | 0 |

**Supplementary table 3** Survivors with neurological symptoms during their ICU stay were invited for an outpatient follow-up examination to explore long-term neurological and neuroimaging findings. Binary variables: 0=no, 1=yes; Abbreviations: CHD=chronic heart disease; HTN=hypertension; OB=obesity; NIC=nicotine; DM=diabetes mellitus; CPD=chronic pulmonary disease; CLD=chronic liver disease; PNIF=pathological neuroimaging findings; VOD=ventilated on discharge; N/A=not applicable, patients did not receive VV-ECMO during their ICU stay.


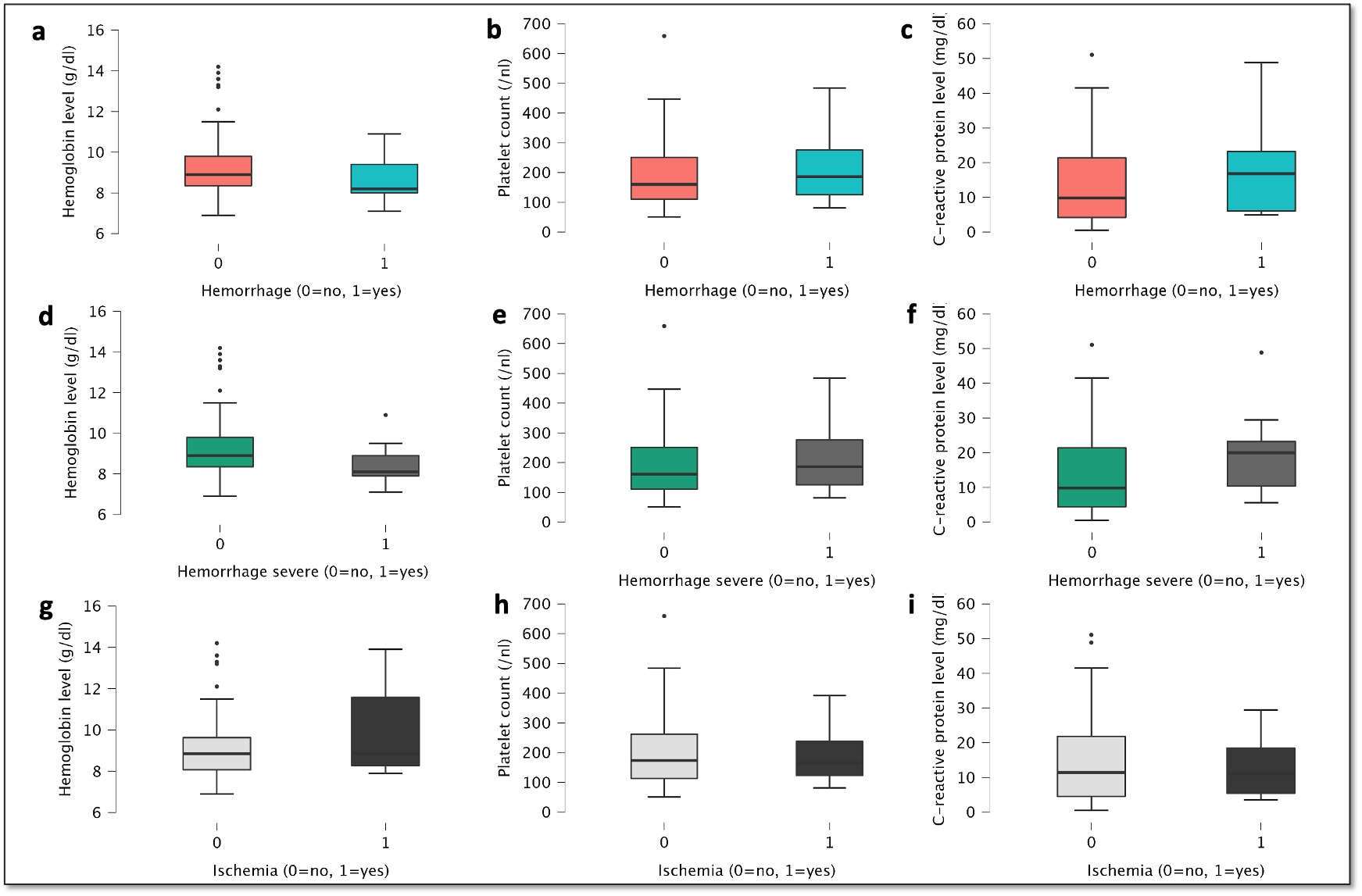


**Supplementary Figure 1** Box and whiskers plot with min, max, 25^th^, 50^th^ (median), 75^th^ percentile and outliers. Employing the Wilcoxon-Mann-Whitney U-test, was no significant difference between patients with and without ischemia or ICH (of any severity and severe) with regard to the following laboratory parameters: Platelet count (/nl), C-reactive protein level (mg/dl) and Hemoglobin level (g/dl).

**Patients with severe microbleeds/siderosis on late follow-up MRI**

Case numbers 6 and 9 had innumerable, punctuate microhemorrhages on late follow-up MRI. Their neurological and neuroimaging findings are depicted in Supplementary figures 2 and 3.


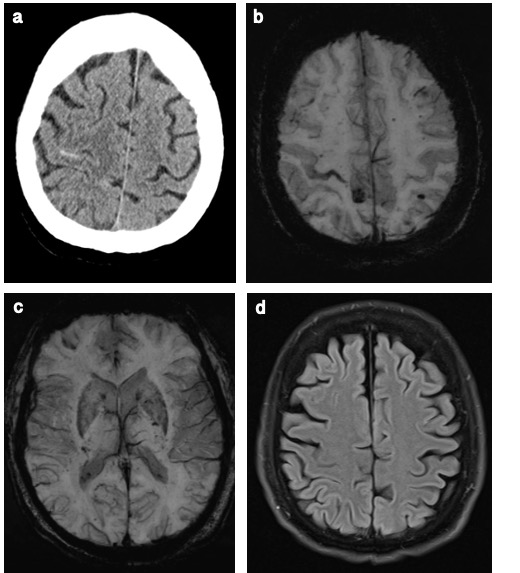


**Supplementary Figure 2 Neuroimaging for Case 6, a 70-year-old male patient.**

**a** Brain CT during the ICU stay, showing atraumatic sulcal subarachnoid hemorrhage in the right frontal lobe and both parietal lobes, and also focal cortical hemorrhage. The CT slice shows sulcal hemorrhage in the right central fissure and focal cortical hemorrhage of the right postcentral gyrus. **b-d** Late follow-up SWI after 238 days showing numerous punctate microhemorrhagic foci within the juxtacortical white matter, of lesser extent in the corpus callosum and bordering the deep gray matter. In addition, it shows siderosis in the right central fissure neighboring the precentral “hand knob” area. The patient was invasively mechanically ventilated for > 40 days but was not in need of VV-ECMO treatment. He received a therapeutic dose of LMWH during his ICU stay which was above the target range at the time point of PNIF. The patient also showed mild cognitive impairment in the MMSE during follow-up examination. He and his wife reported no cognitive impairment prior to his ICU stay. The patient was not impaired with regard to the activities of daily living on the Barthel scale. Neurological examination revealed a positive Romberg’s sign, dysdiadochokinesis, hypoesthesia of the left body half and a distal loss of sensitivity to vibration. Compatible with his symptoms, he stated that his initially diabetic polyneuropathy had worsened during his ICU stay.


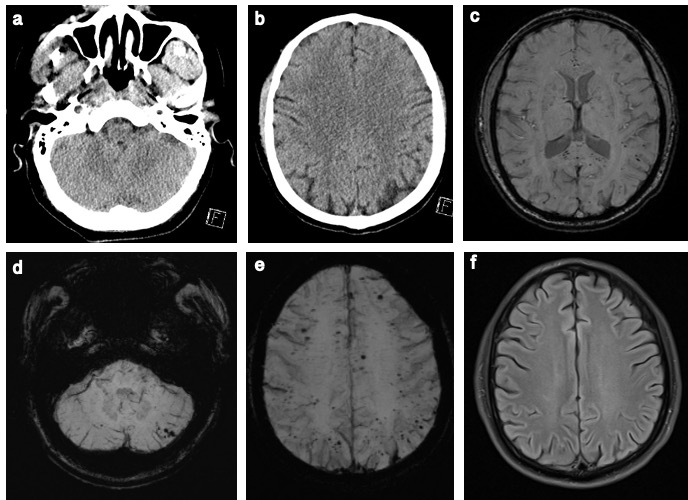


**Supplementary Figure 3 Neuroimaging for Case 9,**

**a 40-year-old male patient. a-b** Brain CT during the ICU stay; even upon retrospective review, this did not reveal punctate microhemorrhages. **c-f** Late follow-up SWI after 172 days showing numerous, punctate microhemorrhagic foci within the juxtacortical white matter, particularly near the depth of the sulci, within the corpus callosum, particularly in the splenium and cerebellar. The patient was invasively mechanically ventilated for > 44 days and with VV-ECMO support for 7 days. He received a therapeutic dose of UFH during his ICU stay which was in the target range. The patient showed no cognitive impairment in the MMSE during follow-up examination and he was not impaired with regard to the activities of daily living on the Barthel scale. Neurological examination revealed a slight, isolated left limb weakness and left facial palsy, an eye tremor, slight dysarthria and dysphagia, a positive Romberg’s sign, dysdiadochokinesis and a distal loss of sensitivity to vibration. These symptoms were compatible with cerebellar ataxia and critical illness polyneuropathy.
